# Harnessing the power of nutritional antioxidants against adrenal hormone imbalance-associated oxidative stress

**DOI:** 10.3389/fendo.2023.1271521

**Published:** 2023-11-30

**Authors:** Anil Patani, Deepak Balram, Virendra Kumar Yadav, Kuang-Yow Lian, Ashish Patel, Dipak Kumar Sahoo

**Affiliations:** ^1^ Department of Biotechnology, Smt. S.S. Patel Nootan Science and Commerce College, Sankalchand Patel University, Visnagar, Gujarat, India; ^2^ Department of Electrical Engineering, National Taipei University of Technology, Taipei, Taiwan; ^3^ Department of Life Sciences, Hemchandracharya North Gujarat University, Gujarat, India; ^4^ Department of Veterinary Clinical Sciences, College of Veterinary Medicine, Iowa State University, Ames, IA, United States

**Keywords:** adrenal hormone imbalance, oxidative stress, nutritional antioxidants, reactive oxygen species, HPT axis

## Abstract

Oxidative stress, resulting from dysregulation in the secretion of adrenal hormones, represents a major concern in human health. The present review comprehensively examines various categories of endocrine dysregulation within the adrenal glands, encompassing glucocorticoids, mineralocorticoids, and androgens. Additionally, a comprehensive account of adrenal hormone disorders, including adrenal insufficiency, Cushing’s syndrome, and adrenal tumors, is presented, with particular emphasis on their intricate association with oxidative stress. The review also delves into an examination of various nutritional antioxidants, namely vitamin C, vitamin E, carotenoids, selenium, zinc, polyphenols, coenzyme Q10, and probiotics, and elucidates their role in mitigating the adverse effects of oxidative stress arising from imbalances in adrenal hormone levels. In conclusion, harnessing the power of nutritional antioxidants has the potential to help with oxidative stress caused by an imbalance in adrenal hormones. This could lead to new research and therapeutic interventions.

## 1 Introduction

Adrenal hormone imbalance or dysfunction refers to a condition characterized by aberrant production or regulation of hormones such as cortisol, aldosterone, and dehydroepiandrosterone (DHEA) inside the body. The presence of this imbalance can significantly impact various physiological processes, resulting in a diverse array of health complications (1). Oxidative stress, which occurs when the body’s antioxidant defence systems cannot neutralize reactive oxygen species (ROS), is one of the main causes of these negative effects (abnormal production or regulation of hormones such as cortisol, aldosterone, and DHEA) (2). A study with human cells found that too much glucocorticoid causes too much ROS to be made, which disturbs the balance of metabolic processes and changes the way the vascular endothelium looks and works (3).

Endogenous antioxidant systems control ROS, chemically reactive molecules produced by cellular metabolism (4). However, when there is an imbalance in the adrenal hormones, this delicate balance is disturbed, which results in increased ROS generation and reduced antioxidant defences (5). The aforementioned imbalance may arise due to factors such as chronic anxiety, hormone dysregulation, environmental pollutants, and suboptimal dietary selections (6). Through increased mitochondrial respiration and oxidative phosphorylation, glucocorticoids directly cause oxidative stress in neurons. The incubation of cortical neurons with acute corticosterone resulted in a dose- and time-dependent increase in mitochondrial oxidation, membrane potential, and calcium-holding capacity (7).

Oxidative stress caused by an imbalance in the adrenal hormones has many effects. Oxidative stress can damage lipids, proteins, and DNA, which can cause cellular dysfunction and tissue damage (5). Furthermore, it can turn on inflammatory pathways and mess up the complex signaling networks needed to keep physiology in balance (8). Consequently, adrenal hormone-related diseases like adrenal insufficiency and Cushing’s syndrome often show signs of oxidative stress, like fatigue, immune dysfunction, cognitive impairment, and accelerated aging (9).

A crucial part of physiological balance is the complicated relationship between antioxidants in the diet and oxidative stress-induced adrenal hormone imbalance (10). The finely tuned regulation of adrenal hormones can be disrupted by oxidative stress, which is caused by an imbalance between reactive oxygen species and the body’s antioxidant defense mechanisms (2). Antioxidants serve an important role in preventing oxidative damage by neutralizing free radicals and protecting the delicate equilibrium of the adrenal glands. Adrenaline hormones such as cortisol and adrenaline, which are essential for stress response and overall hormonal harmony, may be dysregulated when this balance is disrupted. Nutritional antioxidants are bioactive substances found in different foods that can eliminate ROS and boost the body’s own antioxidant defences. Some of these molecules are vitamins (like C and E), minerals (like selenium and zinc), phytochemicals (like polyphenols and carotenoids), and other parts of food (11).

The primary objective of this review is to elucidate the mechanisms by which adrenal hormone imbalance induces oxidative stress and investigate the potential contributions of nutritional antioxidants in mitigating such imbalances. Understanding the intricate interplay between adrenal hormone imbalance, oxidative stress, and nutritional antioxidants can give novel insights regarding therapeutic modalities for disorders associated with adrenal hormones, thereby enhancing holistic well-being.

## 2 Overview of the adrenal gland

**Figure 1:**
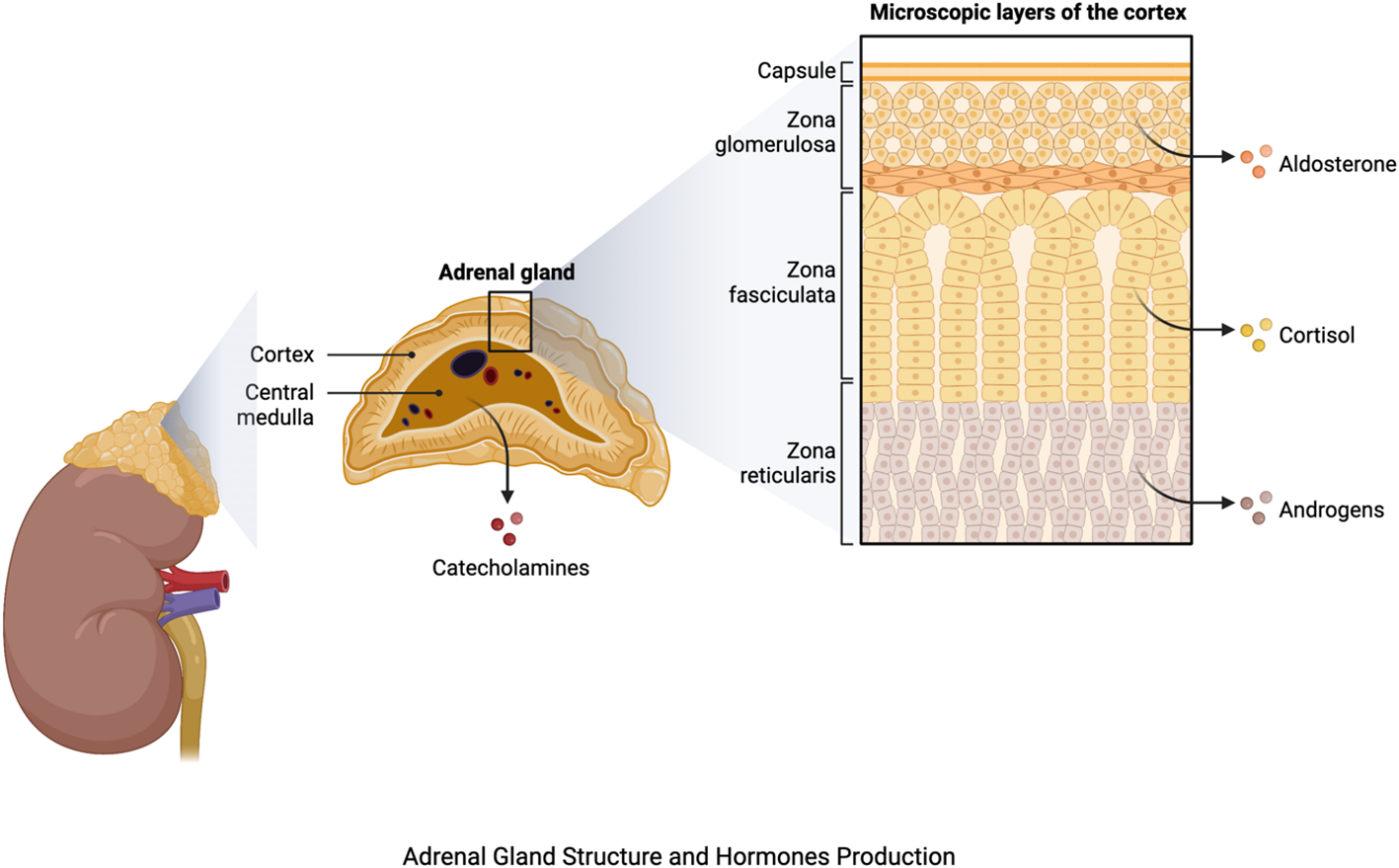
Adrenal gland structure and hormone production. The figure was produced with BioRender (Biorender.com; accessed on 29^th^ July 2023).

The adrenal glands, which are located atop each kidney, are important components of the endocrine system, playing a key role in maintaining homeostasis and responding to stress. Each adrenal gland is divided into two sections: the outer adrenal cortex and the inner adrenal medulla (12). The adrenal cortex is further subdivided into three zones, each of which is responsible for the production of a distinct hormone. Mineralocorticoids, primarily aldosterone, are produced by the outermost zona glomerulosa and regulate electrolyte balance and blood pressure. The zona fasciculata produces glucocorticoids, particularly cortisol, which are involved in glucose metabolism, anti-inflammatory responses, and stress management. Androgens are produced by the innermost zona reticularis, which aids in the development of secondary sexual characteristics (13) (**Figure 1**).

The adrenal medulla, an extension of the sympathetic nervous system, on the other hand, produces catecholamines such as epinephrine (adrenaline) and norepinephrine (14) (**Figure 1**), which have widespread impacts on the cardiovascular system, metabolism, and other body systems. They cause the bloodstream to release glucose and fatty acids, priming the body for increased activity. Furthermore, these hormones increase bronchiole dilation, resulting in enhanced oxygen uptake (15). The hormones produced by the adrenal glands are many and diverse, regulating a wide range of physiological functions. Aldosterone influences blood pressure and electrolyte balance through regulating sodium and potassium levels (16). Cortisol has an impact on metabolism, immunological function, and the body’s reaction to stress. Androgens play a role in the development of secondary sexual characteristics in men (17).

Furthermore, the adrenal glands produce dehydroepiandrosterone (DHEA) and its sulfate (DHEA-S), which are precursors of sex hormones that influence sexual development and reproductive function. The interplay of these hormones is complex, regulating a wide range of physiological processes and contributing to the body’s ability to adapt to both short-term and long-term stressors (18).

## 3 General overview of adrenal hormone disorders

Adrenal hormone disorders, also called adrenal gland disorders, are characterized by dysfunction or imbalance in the hormones produced by the adrenal glands. The adrenal glands are located on top of the kidneys and are responsible for producing hormones that regulate numerous physiological processes. Common adrenal hormone disorders include adrenal insufficiency, Cushing’s syndrome, and adrenal tumors (19).

### 3.1 Adrenal insufficiency

When the adrenal glands do not produce enough cortisol and, occasionally, aldosterone, it is known as adrenal insufficiency (20). Adrenal insufficiency is a common disorder with multiple causes that can be categorized as primary (adrenal), secondary (pituitary), and tertiary (hypothalamus) forms (21). Primary adrenal insufficiency, often called Addison’s disease, is mostly caused by autoimmune adrenal gland damage, but infections and genetic abnormalities can also contribute. Primary adrenal insufficiency is characterized by fatigue, frailty, weight loss, low blood pressure, salt cravings, and skin hyperpigmentation (22). When the pituitary gland is unable to produce enough adrenocorticotropic hormone, which stimulates the adrenal glands to synthesize cortisol, secondary adrenal insufficiency develops (23). Tertiary adrenal insufficiency caused by exogenous steroid medication is common but difficult to diagnose due to its non-specific symptoms (21).

### 3.2 Cushing’s syndrome

Long-term cortisol exposure causes Cushing’s syndrome. Exogenous Cushing’s syndrome is caused by corticosteroid use, while endogenous is caused by adrenal gland excessive production of cortisol (24). The etiology of endogenous Cushing’s syndrome encompasses various factors, including the presence of adrenal tumors (adenomas or carcinomas), pituitary tumors (Cushing’s disease), or tumors that generate ACTH elsewhere in the body (25). Cushing’s syndrome is linked to severe morbidities and a higher mortality rate. Cardiovascular disease is the leading cause of systemic complications and the leading cause of mortality. The prognosis of the disease is primarily influenced by the diagnostic and therapeutic difficulties that continue to be a significant obstacle (26). Weight gain (especially in the trunk and face), muscle weakness, thinning skin, easily bruising, elevated blood pressure, glucose intolerance, and mood swings are all typical signs of Cushing’s syndrome (27).

### 3.3 Adrenal tumors

Tumors of the adrenal glands can be benign (noncancerous) or malignant (cancerous). Adrenal adenomas are the most prevalent adrenal tumor type and are typically nonfunctional, meaning they do not produce excessive hormones (28). Specific hormones, such as cortisol (which causes Cushing’s disease) or aldosterone (which causes primary aldosteronism), can be overproduced as a result of functional adrenal tumors (29). Rare but aggressive malignant tumors known as adrenal carcinomas can produce too much hormone and invade adjacent tissues (30). Clinical assessment, hormone level measurements (such as cortisol, aldosterone, and adrenal androgens ACTH), imaging tests (such as CT scan and MRI) to look for abnormalities in the adrenal glands, and occasionally specialized tests like the dexamethasone suppression test or adrenal vein sampling are used to diagnose adrenal hormone disorders (31). The exact illness and its underlying cause will determine the available treatments. They could include radiation therapy, surgery to remove adrenal tumors, hormone replacement therapy (such as cortisol or aldosterone replacement), or drugs to control symptoms or hormone production (28).

### 3.4 Adrenal steroidogenesis

The pathophysiology of lethal adrenal disorders is heavily influenced by oxidative stress, and mutations in antioxidant defense genes can have a considerable impact on adrenal steroidogenesis. Because of their high metabolic activity, the adrenal glands, which are required for the production of steroid hormones that regulate different physiological processes, are vulnerable to oxidative damage (32). Excessive oxidative stress can disrupt adrenal steroidogenesis by disrupting key enzymes involved in hormone synthesis, resulting in cortisol and aldosterone production dysregulation. Mutations in antioxidant defense genes, which are important for reducing oxidative damage, worsen this sensitivity. Such genetic variants weaken cellular defense mechanisms against ROS, raising oxidative stress levels in the adrenal glands. This complex interplay between oxidative stress and genetic factors might lead to the emergence and progression of life-threatening adrenal disorders (33).

## 4 Oxidative stresses

Oxidative stress occurs when prooxidant molecules like ROS and reactive nitrogen species (RNS) get produced in excess when antioxidant systems are not working efficiently (34, 35). The mitochondrial respiratory chain produces superoxide anion, hydroxyl radical, and hydrogen peroxide during aerobic metabolism (36). RNS includes peroxynitrite-nitrosoperoxycarbonate is produced when peroxynitrite and carbon dioxide react with nitric oxide (NO) (37). Under normal physiological settings, the body makes ROS as a byproduct of metabolism and other cellular processes. Though ROS are important for cell signaling, immune function, and defence against pathogens (38), oxidative stress can be produced by either excessive ROS generation or insufficient antioxidant defence mechanisms (2). Environmental pollutants, exposure to ionizing radiation, certain drugs, chronic inflammation, and lifestyle choices such as excessive alcohol use, a poor diet, and smoking can all contribute to oxidative stress (39). In addition, elevated oxidative stress has been associated with several diseases and conditions, including diabetes, neurological disorders, cardiovascular diseases, and cancer (40).

When ROS levels exceed the antioxidant defences of the body, they can damage lipids, proteins, and DNA. This is also known as oxidative damage, and it can interfere with normal cellular function and contribute to the development of numerous diseases and aging processes (41). This is also linked to Alzheimer’s, Parkinson’s, cardiovascular diseases, and cancer (42). ROS play a major role in disease development, including cancer, neurodegenerative diseases, cardiovascular diseases, diabetes, and inflammatory diseases. The production of ROS is increased in obesity due to the metabolic burden imposed by excessive macronutrient intake and the availability of substrates (43). Metabolic perturbations in the adipose tissue of individuals with obesity arise as a consequence of mitochondrial dysfunction and endoplasmic reticulum stress within the cellular milieu (44). The accumulation of ROS leads to cellular impairment and subsequently contributes to the pathogenesis of inflammatory and cardiovascular disorders (45). The communication of pro-inflammatory cytokines by mitochondrial ROS serves to reinforce the relationship between OS and inflammation (46).

The human body employs a sophisticated array of antioxidants to combat oxidative stress. These include enzymatic antioxidants such as superoxide dismutase (SOD), catalase, glutathione peroxidase, and non-enzymatic antioxidants, including glutathione (GSH). Antioxidants have the ability to counteract ROS and mitigate cellular damage caused by oxidative stress (47).

The management of oxidative stress is crucial for the maintenance of overall health. A balanced, nutritious diet high in antioxidants, regular exercise, avoiding exposure to environmental toxins, and reducing lifestyle factors known to reduce oxidative stress, whereas smoking and binge drinking, are all effectively increase oxidative stress. Additionally, it’s crucial to note that some antioxidant supplements have been investigated for their possible advantages in lowering oxidative stress, while the data for their efficiency is conflicting and should be reviewed with a healthcare provider (39, 48).

## 5 Adrenal hormonal imbalance-associated oxidative stress

Adrenal hormonal imbalance-related oxidative stress is caused by dysregulation in the finely tuned endocrine system, specifically the adrenal glands (49). The adrenal glands are vital for maintaining physiological homeostasis by secreting hormones such as cortisol and adrenaline. When this equilibrium is upset, either by chronic stress or pathological situations, it can result in the overproduction or underproduction of these hormones, which contributes to oxidative stress (50).

Excess cortisol release, which is frequently associated with chronic stress, activates the glucocorticoid receptor, boosting the creation of reactive oxygen species (ROS) within cells. These ROS molecules, which include superoxide and hydrogen peroxide, cause oxidative damage to cells by destroying lipids, proteins, and DNA (51). Furthermore, disturbed hormonal balance changes antioxidant defense mechanisms, worsening oxidative stress (52).

The mitogen-activated protein kinase (MAPK) and nuclear factor-κB (NF-κB) pathways have both been associated in adrenal hormonal imbalance-associated oxidative stress. When activated by stress-induced hormonal imbalances, these pathways promote the production of pro-inflammatory cytokines and genes associated with oxidative stress, increasing the overall oxidative burden on cells (53). Furthermore, the hypothalamic-pituitary-adrenal (HPA) axis, a key component in stress response, plays an important role in adrenal hormonal imbalance. Abnormal HPA axis signaling can cause persistent cortisol increase, causing oxidative stress via many mechanisms, including mitochondrial dysfunction and endoplasmic reticulum stress (54). The adrenal glands generate hormones such as glucocorticoids, mineralocorticoids, and androgens, (**Figure 1**) which play significant roles in regulating various physiological processes (55).

The **Table 1** provides a brief overview of the impact of hormonal imbalances on oxidative stress, with a focus on glucocorticoids, mineralocorticoids, and androgens. In the case of glucocorticoids, both excess (Cushing’s syndrome) and insufficiency (Addison’s disease) contribute to oxidative stress through a variety of mechanisms, such as decreased antioxidant defenses, increased reactive oxygen species (ROS) generation, and impaired mitochondrial function, which ultimately results in chronic inflammation. Mineralocorticoids, whether in excess (Hyperaldosteronism) or in deficiency (Hypoaldosteronism), are linked to oxidative stress, mainly via activation of the renin-angiotensin-aldosterone system (RAAS) and disruption of cellular homeostasis due to sodium and potassium imbalances. Androgens, whether in excess (Hyperandrogenism) or in deficiency (Hypoandrogenism), cause oxidative stress by affecting immunological responses, fostering chronic inflammation, causing mitochondrial dysfunction, and causing oxidative damage in reproductive organs, thereby affecting fertility and reproductive health (**Table 1**).

**Table 1:** Adrenal hormonal imbalance-associated oxidative stress.

Hormone	Imbalance	Effects on Oxidative Stress	Reference
Glucocorticoids	Excess (Cushing’s syndrome) or deficiency (Addison’s disease)	- Reduced antioxidant defenses: decreased endogenous antioxidant synthesis (e.g., glutathione, SOD, catalase). - Increased ROS generation: Activation of NADPH oxidase stimulates ROS production (superoxide anions, hydrogen peroxide). - Impaired mitochondrial function: Impairment of mitochondrial activity, which results in increased ROS generation within the mitochondria. - Inflammation and oxidative stress: Immune system imbalance and promotion of chronic inflammation, linked to increased oxidative stress.	(33)
Mineralocorticoids	Excess (Hyperaldosteronism) or deficiency (Hypoaldosteronism)	- Excess aldosterone can lead to increased activation of the renin-angiotensin-aldosterone system (RAAS), which is in relation with oxidative stress. - Mineralocorticoid imbalances can disrupt sodium and potassium balance, disrupting cellular homeostasis and causing oxidative stress indirectly.	(56)
Androgens	Excess (Hyperandrogenism) or deficiency (Hypoandrogenism)	- Inflammation and oxidative stress: An imbalance in androgen levels can alter the immunological response and contribute to chronic inflammation, both of which lead to increased oxidative stress. - Mitochondrial dysfunction: Changes in testosterone levels can impair mitochondrial function and increase ROS generation, leading to oxidative stress. - Excess androgen levels can cause oxidative damage to reproductive organs, impacting fertility and reproductive health.	(57)

### 5.1 Glucocorticoids hormone imbalance -associated oxidative stress

In the brain, lungs, and blood cells, the association between glucocorticoids and oxidative stress has been established. Numerous inflammatory and autoimmune disorders are frequently treated with glucocorticoids. The glucocorticoids’s excess led to myopathy, osteoporosis, diabetes, and hypertension, among other diseases. All of the previously mentioned pathophysiological conditions are linked to oxidative stress. ROS from glucocorticoids can cause many pathological conditions (58). Dexamethasone, a synthetic glucocorticoid, has been documented to elicit the production of ROS either through direct means or as a consequence of endothelial nitric oxide synthase (eNOS) uncoupling, which is attributed to the constrained availability of tetrahydrofolate (59, 60). When glucocorticoids are produced in excess, they cause glucose levels to rise; this leads to glycation, which increases ROS production; this, in turn, reduces catalase (CAT), glutathione peroxidase (GPx), and SOD levels in the hippocampus, impairing cognitive functions (61). By raising mitochondrial respiration and oxidative phosphorylation, glucocorticoids directly cause neuronal OS (62). A cascade of negative effects is associated with the role of adrenal corticosterone in hippocampus oxidative stress. This disease is characterized by increased lipid peroxidation and protein carbonyl (PC) concentrations, as well as a decrease in antioxidant enzyme activity such as GPx, SOD, and CAT (63). Another study found that short-term exogenous cortisol administration did not enhance juvenile brown trout oxidative stress levels but did increase GSH levels, indicating that the increased GSH may have reduced the formation of ROS (64). Therefore, cortisol may prevent rather than cause oxidative stress and may activate antioxidant defences via genomic pathways in addition to influencing other systems that regulate the formation of pro-oxidants like ROS (65).

Familial glucocorticoid deficiency (FGD) arises due to mutations in the ACTH-receptor components (MC2R, MRAP) or the general steroidogenesis protein (StAR). These mutations lead to an inability of the adrenocortical cells to synthesize glucocorticoids in response to ACTH stimulation. *Nicotinamide Nucleotide Transhydrogenase (NNT)* mutations are responsible for the development of FGD. These mutations were observed to decrease the ability of adrenocortical cells to effectively detoxify ROS. Mutations in NNT result in the manifestation of OS as well as phenotypic and functional abnormalities in mitochondrial activity. These findings provide compelling evidence supporting the important role of NNT in maintaining proper mitochondrial function in cases of adrenocortical insufficiency (66).

### 5.2 Mineralocorticoids hormone imbalance-associated oxidative stress

According to numerous clinical and investigations in animal models, the most significant physiological mineralocorticoid, aldosterone, causes OS and inflammation in patients with chronic and stable heart failure (67). Aldosterone raises blood pressure, affecting the heart. Mineralocorticoid receptors directly affect cardiac function, electrical conduction, OS, inflammation, and fibrosis, further harming the heart (68). Aldosterone/salt-induced hypertension in rats results in renal damage and an increase in the production of ROS in the renal cortex (69). According to Patni et al. (70) findings, an increase in renal OS causes the induction of apoptosis in the renal tubules. It has been demonstrated that aldosterone stimulates superoxide radical formation in endothelial cells by activating Rec1 (71). The mineralocorticoid receptor (MR) is activated during both physiological and pathological events since tissue damage, OS, and inflammation are frequent components of disease situations. It has been utilized in clinical trials to treat heart and kidney disease connected to hypertension and other chronic diseases by blocking MR signaling with MR antagonists (MRAs), which suppresses fibrosis in these organs as MRAs likely have cardio-protective effects by directly blocking cardiac and vascular MR (72, 73).

To avoid aldosterone-induced oxidative damage, kidney cells were tested for their potential to up-regulate nuclear factor-erythroid-2-related factor 2 (Nrf2) (74). Aldosterone first activated Nrf2 *in vitro* as an antioxidant response. Although aldosterone-induced oxidative or nitrative stress quickly stimulated antioxidant or detoxifying enzymes such SOD, thioredoxin (TRX), HO-1, or GCSc, this adaptive survival response appeared to be fleeting and overpowered by a chronic increased generation of ROS/RNS. As a result, oxidative DNA damage happened. Additionally, even though Nrf2 activation was seen *in vivo*, aldosterone-treated rat kidneys showed significant DNA damage, showing that the response was insufficient to shield the animals from these side effects (75).

### 5.3 Androgens hormone imbalance-associated oxidative stress

Testosterone, the main male steroid hormone, causes spermatogenesis and secondary sexual characteristics. Testosterone is anabolic. Usually, it speeds up metabolism. Increased metabolic rate increases O_2_ consumption and ROS generation. Thus, testosterone increases OS. However, testosterone’s role in OS is controversial. Multiple studies have demonstrated that testosterone induces OS in the muscle, testis, and human placenta (76); others indicate that testosterone has antioxidant properties in the prostate and nervous tissue (77). Testosterone supplements improve the OS parameters in brain tissues and raise antioxidant enzyme levels to reduce oxidative damage. According to *in vitro* research, testosterone treatment in newborn rats specifically protects the cerebellar granule cells from OS-induced cell death. By inhibiting OS, testosterone contributes to the protection of neurons (78). Adrenal androgen imbalances, such as dehydroepiandrosterone (DHEA) and androstenedione, have been linked to oxidative stress in the body. Elevated amounts of these androgens, which are commonly found in conditions such as polycystic ovarian syndrome (PCOS) and some adrenal disorders, can upset the delicate balance of cellular redox processes. This imbalance can result in an excess of reactive oxygen species (ROS), which then overwhelms the body’s antioxidant defences, resulting in oxidative stress. This type of oxidative stress is recognized as a key factor in the pathogenesis of a variety of health problems, ranging from metabolic disorders to inflammation-related diseases, emphasizing the importance of understanding and managing adrenal androgen imbalances to mitigate oxidative stress and its associated health consequences (79).

According to, Tam et al. (80) study, androgen deprivation increased ROS anabolism and decreased antioxidant detoxification, which in turn caused OS in the ventral prostate of rats. These researchers discovered that castration caused 4-hydroxynonenal and 8-hydroxy-20-deoxy-guanosine protein adducts in the regressing epithelium, which suggests oxidative damage. Additionally, castration considerably decreased the expression of important antioxidant enzymes (AOEs) (GPx1, thioredoxin, SOD2, and peroxiredoxin 5) and markedly increased the expression of ROS-generating NAD(P)H oxidases. Testosterone supplementation partially repaired oxidative damage in ventral prostate epithelia of castrated rats receiving testosterone replacement therapy had a partial decrease in NAD(P)H oxidase expression but an increase in GPx1, SOD2, peroxiredoxin 5 expression, thioredoxin, CAT, glutathione reductase (GR), c-glutamyl transpeptidase, and glutathione synthetase expression in the regenerating ventral prostate tissue. The augmentation of mitochondrial functionality, alterations in intracellular GSH concentrations, and elevation of c-glutamyl transpeptidase activity collectively facilitated the physiological amplification of androgens, leading to the potentiation of OS in LNCaP human prostate cancer cells that are responsive to androgens (81). Prasad et al. (82) showed that testosterone injection to mice caused the down-regulation of CAT, SOD, GST, and GR in the prostate gland. Androgens lowered the activity of AOEs in the heart of rats (83), while orchidectomy enhanced aortic Cu/ZnSOD (84). Borst et al (85) found that testosterone supplementation significantly increased the revival of cardiac work following ischemia/reperfusion *in vitro*, while orchidectomy significantly lowered rat left ventricular AOE activities. In contrast, Klapcinska et al. (86) showed that castration of male rats lowered the levels of CAT, SOD, GPx, and GR in the left ventricle of the heart, as well as GSH and protein-thiol groups, and increased lipid peroxidation and nitrotyrosine concentrations. Increases in a- and c-tocopherol tissue concentrations in the left ventricle appeared to be a compensatory reaction to the increased OS brought on by gonadectomy. Although androgen replacement restores a healthy serum testosterone level, it decreases left ventricular tissue antioxidant status. The favorable effect of endurance training on SOD and CAT activities was reversed, and myocardial lipid peroxidation was enhanced in adolescent male Wistar rats administered with a high dosage of testosterone (87).

Dehydroepiandrosterone levels gradually decline with age. It has been suggested that DHEA is an effective ageing index with links to geriatric syndromes. There is evidence that low DHEA levels are linked to the beginning and progression of metabolic syndrome and diabetes mellitus, as well as a decrease in bone mineral density (88, 89). Numerous studies have revealed that markers in the oxidative circulation are higher than normal in PCOS patients and that oxidative stress plays a role in the development of PCOS (90). In PCOS, hyperglycemia-induced oxidative stress may be capable of directly promoting hyperandrogenism. The link between plasma testosterone or androstenedione and ROS production suggests this. *In vitro* investigations have revealed that the ovarian steroidogenic enzymes responsible for androgen production are stimulated by oxidative stress and inhibited by antioxidants such as statins (91, 92).

## 6 Role of nutritional antioxidants in alleviating adrenal hormone imbalance

**Figure 2:**
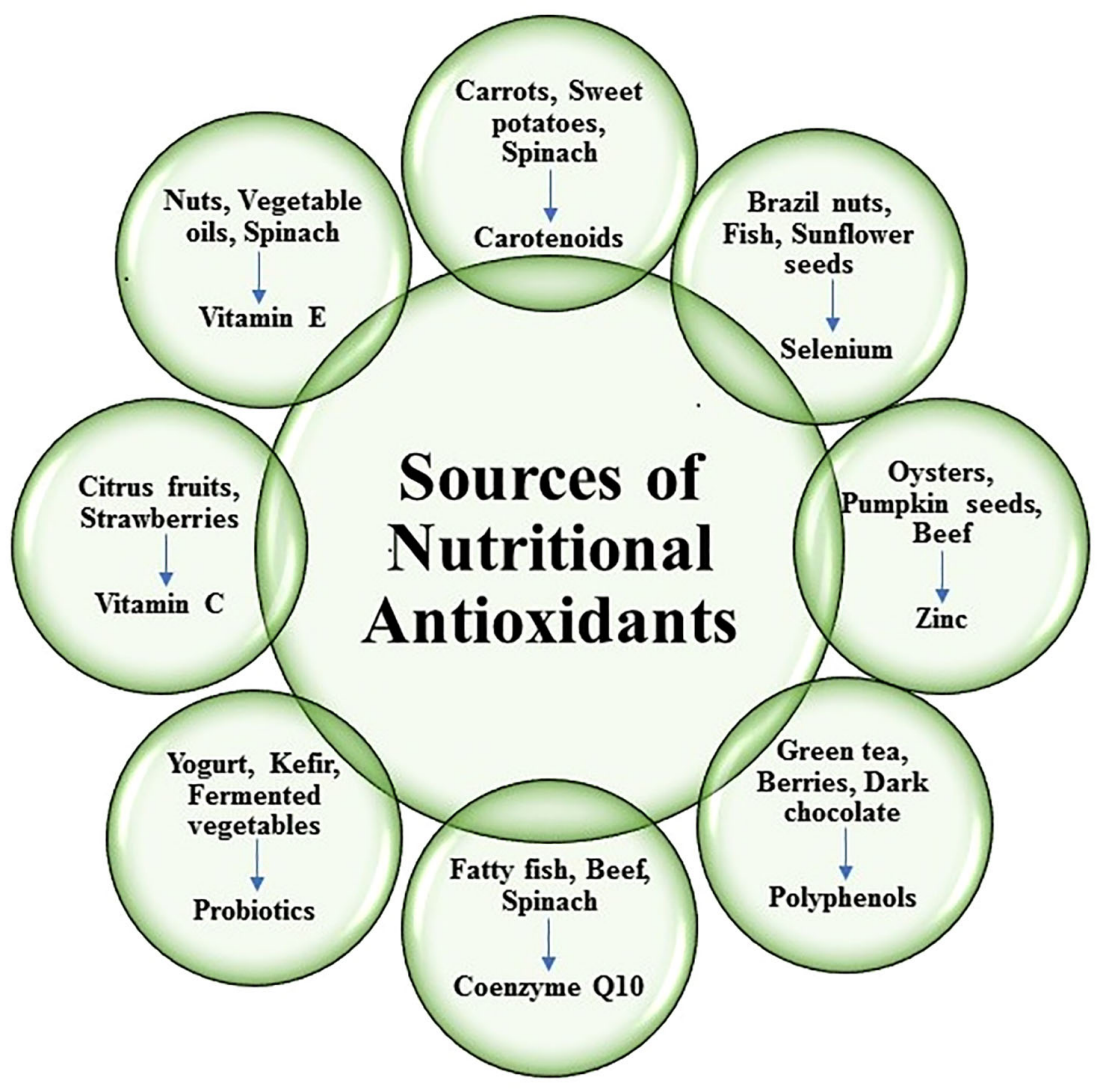
Sources of different types of nutritional antioxidants.

**Figure 3:**
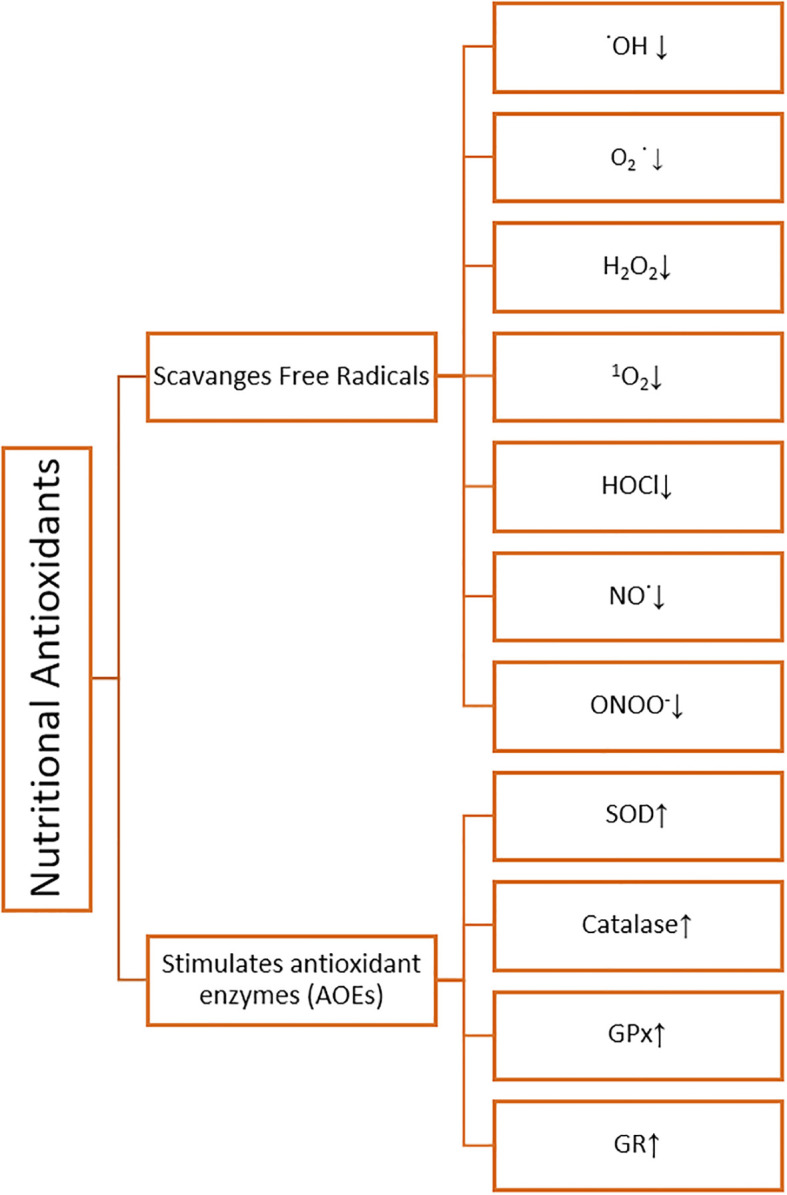
Schematic representation of nutritional antioxidant effects.

Nutritional antioxidants are substances that can be found in a variety of foods and are essential in defending the body against free radical damage, oxidative stress, and the negative impact of reactive oxygen species (ROS). They function by scavenging or neutralizing these harmful chemicals, reducing cellular damage, and improving general health (71). Different types of nutritional antioxidants are depicted in (**Figure 2**). It was revealed to scavenge hydroxyl radical (˙OH), superoxide anion radical (O_2_˙^-^), hydrogen peroxide (H_2_O_2_), hypochlorous acid (HOCl), nitric oxide (NO˙), peroxynitrite (ONOO^-^), singlet oxygen (^1^O_2_) and stimulates antioxidant enzymes (AOEs) SOD, Catalase, GPx, GR by nutritional antioxidants (59, 93) (**Figure 3** **).**

### 6.1 Vitamin C against adrenal hormone imbalance-associated oxidative stress

Water-soluble vitamin C, also referred to as ascorbic acid, is a potent antioxidant. Essential for the preservation of overall well-being, it is imperative due to its involvement in numerous pivotal physiological processes (94). Free radicals are unsteady molecules that can harm cells, resulting in several health issues and hastening aging. Vitamin C functions as an antioxidant that assists in scavenging these free radicals and lowering oxidative stress (95). Vitamin C is required for the production of collagen, a structural protein found in the epidermis, bones, tendons, and blood vessels. Collagen is necessary for the formation of scar tissue, so it plays a crucial role in wound healing. Additionally, vitamin C promotes healthy gums, teeth, and cartilage (96). It is well-known that vitamin C supports immune function. It promotes the production of white blood cells, which are essential for fending off infections and pathogens. It enhances natural killer cells and immune system function (97). Plant-based diets and iron supplements contain non-heme iron, which is better absorbed with vitamin C. Iron deficiency anemia can be prevented by boosting iron absorption with vitamin C (98). Other antioxidants in the body, such as vitamin E, are also renewed by vitamin C. It facilitates the restoration of the antioxidant capacity of vitamin E, which enables it to continue fulfilling its protective function in cell membranes. Citrus fruits like lemons, oranges, and grapefruits, berries like strawberries, kiwi, pineapple, mango, papaya, bell peppers (especially yellow and red), Brussels sprouts, broccoli, and leafy greens like spinach and kale are all excellent sources of vitamin C. Furthermore, achieving this outcome is feasible by administering vitamin C supplements (99).

Excess cortisol production in circumstances such as Cushing’s disease or persistent stress can contribute to increased oxidative stress in the body. Vitamin C can help reduce the oxidative stress consequences caused by adrenal hormone imbalance (100).

Vitamin C is a powerful antioxidant capable of scavenging and neutralizing reactive oxygen species (ROS) produced during oxidative stress. It helps stabilize free radicals by giving electrons, preventing them from causing harm to cellular components (95). Vitamin C is essential for regenerating other antioxidants such as vitamin E, glutathione, and coenzyme Q10. These antioxidants also aid in the reduction of oxidative stress and the maintenance of a healthy cellular environment (47). Vitamin C is required for the manufacture of collagen, a protein that provides structure as well as support to a variety of tissues throughout the body, including the adrenal glands. Vitamin C promotes collagen synthesis, which aids in the integrity and function of the adrenal glands, potentially lowering the risk of hormone imbalances. Chronic oxidative stress caused by adrenal hormone abnormalities may weaken the immune system (101). Vitamin C improves immune function by promoting the growth and activity of immune cells like lymphocytes and phagocytes. This can assist the body in fighting infections and other immune-related problems (97). Vitamin C has been demonstrated to influence the body’s stress response. It aids in the regulation of cortisol production, the principal stress hormone generated by the adrenal glands. Vitamin C may indirectly assist in minimizing oxidative stress associated with chronic stress by maintaining adrenal health and normalizing cortisol levels (100).

**Figure 4:**
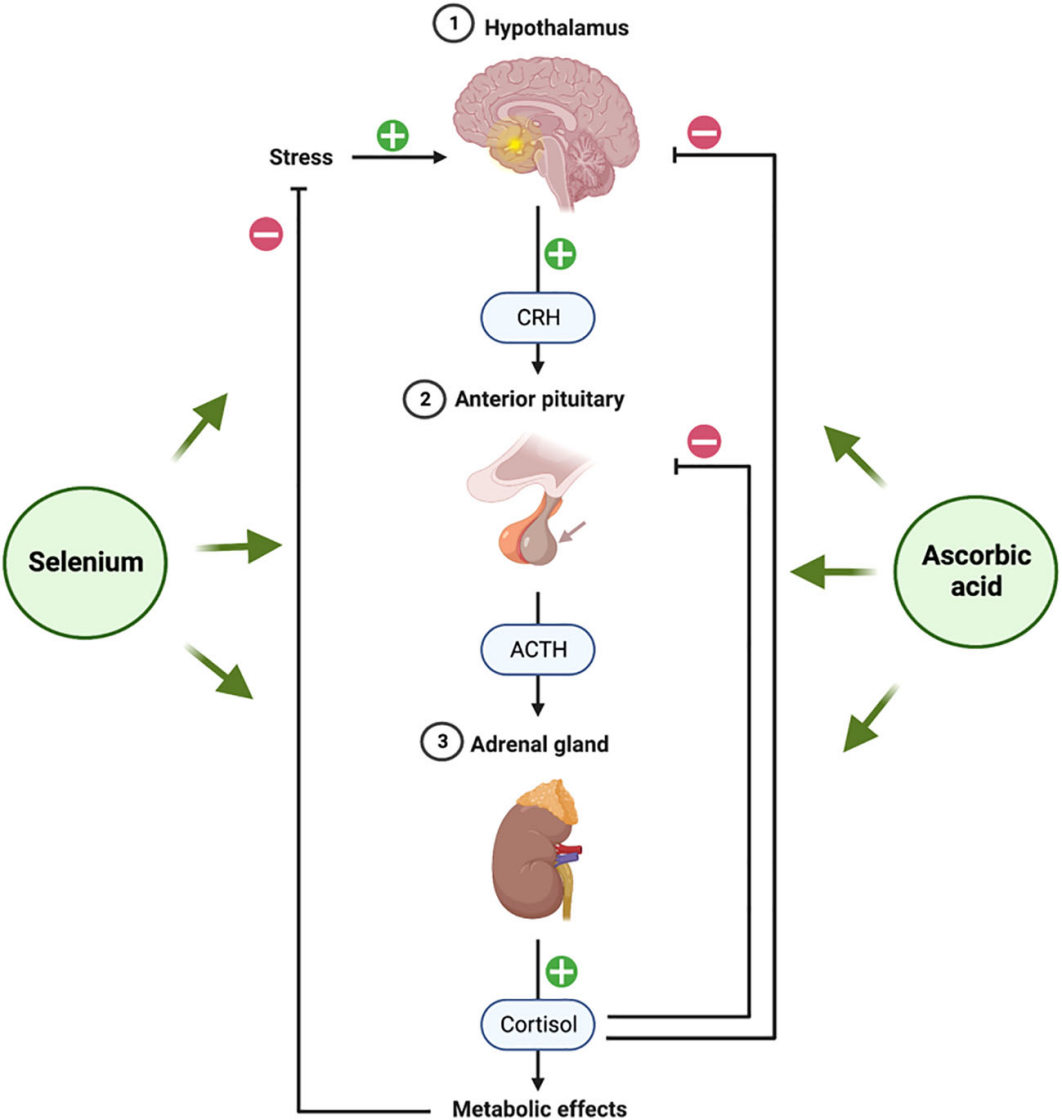
Hypothalamic-Pituitary-Adrenal (HPA) axis and role of selenium and ascorbic acid supplements. The figure was produced with BioRender (Biorender.com; accessed on 29^th^ July 2023).

Several studies have revealed a link between stress-related behavior and ascorbic acid. Animal studies show that ascorbic acid reduces stress-induced cortisol production. Ascorbic acid modulates the hypothalamic-pituitary-adrenal (HPA) axis (**Figure 4**) by directly “braking” cortisol secretion (102). Since ascorbic acid is a cofactor for adrenal cortex enzymes involved in glucocorticoid biosynthesis, this vitamin is necessary for its production (103). By acting as a cofactor for 11β-hydroxylase, for instance, the ascorbate somewhat increases the conversion of 11-deoxycortisol to cortisol and keeps the cortisol tone at physiological levels (104). Ascorbic acid appears to play a significant part in the stress response, as evidenced by the high amounts of ascorbic acid found in the adrenal glands and the production of ascorbic acid in response to ACTH (100). This is supported by studies showing that ascorbate release occurs before corticosteroid release in the adrenal gland after systemic administration of ACTH to hypophysectomized rats (105), a finding that suggests ascorbate must first be released by the adrenal gland for steroid synthesis (or release) to begin when there is stress.

### 6.2 Vitamin E against adrenal hormone imbalance-associated oxidative stress

A class of fat-soluble substances with antioxidant capabilities is referred to as vitamin E. It can be found in seeds, leafy green vegetables, nuts, and vegetable oils. There exist two principal variants of vitamin E, namely alpha-tocopherol and gamma-tocopherol. It is worth noting, however, that the term “vitamin E” encompasses a group of eight distinct compounds. These compounds are present in a variety of food sources and are also accessible in the form of dietary supplements (106). Vitamin E antioxidants serve to protect cells against the detrimental effects of free radicals. Free radicals are responsible for inducing cellular damage and contributing to the process of aging. Vitamin E mitigates oxidative stress inside the human body through its ability to counteract the detrimental effects of free radicals (39). Vitamin E is predominantly acknowledged as a lipid-soluble antioxidant, whereby it operates within lipid-rich compartments of cellular structures, such as cell membranes, to mitigate lipid oxidation. Furthermore, it is widely believed that the immune system and gene expression may experience advantageous effects (107).

Adrenal hormone imbalance can increase oxidative stress in the body, and some evidence suggests that vitamin E may protect against it (108). As an antioxidant, Vitamin E neutralizes free radicals and ROS generated during cellular metabolism. Adrenal hormone imbalance can enhance ROS production, causing oxidative damage to cells and tissues. Vitamin E aids in the scavenging of these damaging chemicals, thereby lowering oxidative stress (109). It has been demonstrated that vitamin E affects the body’s hormone levels, especially those of the adrenal glands. Vitamin E may assist in regulating adrenal gland activity and perhaps lessen the production of stress-related chemicals like cortisol by fostering hormonal equilibrium. This may indirectly lessen the oxidative stress brought on by the imbalance of adrenal hormones (110). Chronic adrenal hormone imbalance can cause the body to become inflamed, increasing the risk of oxidative stress. Due to its anti-inflammatory qualities, vitamin E may help lessen inflammation brought on by an imbalance in adrenal hormones. Vitamin E can indirectly lower oxidative stress levels by reducing inflammation (111). Cell membranes are protected from oxidative damage by vitamin E. Unbalanced levels of adrenal hormones can worsen oxidative stress within cells, which can harm biological components. Vitamin E helps maintain appropriate cellular function and lessens oxidative stress-related damage by maintaining the integrity of cell membranes (112).

The consideration of the impact of glucocorticoids on the production of free radicals in the context of stressful situations holds significant importance. Long-term treatment of glucocorticoids is associated with oxidative brain damage in primates, which has been demonstrated (113). According to Al-Sowayan (114), vitamin E therapy reduces exposure to neurotransmitters that cause hypotension by boosting total glutathione, hydrosulfide groups, and selenium levels in the liver and serum. Vitamin E’s chain-breaking antioxidant activities minimize oxidative damage by scavenging free radicals (115).

### 6.3 Carotenoids against adrenal hormone imbalance-associated oxidative stress

A class of pigments known as carotenoids can be found in many different fruits, plants, and other living things. They have been known for being antioxidants and are important for human nutrition (116). In the human body, carotenoids function as antioxidants to help shield cells from injury from harmful compounds known as free radicals. Oxidative stress is a result of free radicals and is linked to several chronic diseases and the aging process. Carotenoids combat these free radicals, minimizing oxidative damage and boosting general health (117). It is worth noting that a mere fraction of the extensive repertoire of carotenoids, exceeding 600 in number, are habitually ingested by people in general. Beta-carotene, lycopene, lutein, zeaxanthin, and astaxanthin are a few well-known carotenoids. Every carotenoid has different antioxidant capabilities and potential health advantages. Many fruits and vegetables contain large amounts of carotenoids. Beta-carotene is abundant in orange and yellow fruits and vegetables like carrots, sweet potatoes, mangoes, and apricots. Watermelons and tomatoes both contain significant levels of lycopene. Zeaxanthin and lutein are found in leafy green vegetables like kale and spinach. Seafood frequently contains astaxanthin, especially salmon and shrimp (118).

Due to their antioxidant action, carotenoids have been linked to many health advantages. Particularly concentrated in the retina, lutein, and zeaxanthin help prevent age-related macular degeneration (AMD) and cataracts. Beta-carotene and astaxanthin, in particular, can help prevent UV damage to the skin and enhance its appearance (119). Carotenoids reduce inflammation and boost immune cell function, boosting immunity. Certain carotenoids, such as lycopene, have been linked to a reduced risk of heart disease by protecting blood vessels from oxidative injury. Carotenoids are absorbed differently depending on food preparation (cooking, chopping, etc.), the presence of dietary lipids, and individual metabolic differences. Carotenoids are more readily absorbed when consumed with a modest amount of fat. In order to exert their full effects, certain carotenoids, such as beta-carotene, must be converted into vitamin A in the body (120).

Carotenoids have been studied for their possible advantages in lowering oxidative stress and promoting general health because of their well-known antioxidant qualities. Although there is little direct study on the benefits of carotenoids directly on oxidative stress related to oxidative stress linked with adrenal hormone imbalance, their antioxidant and anti-inflammatory characteristics may have beneficial impacts (121).

The body uses carotenoids like beta-carotene, lycopene, and lutein as powerful antioxidants. They aid in scavenging dangerous reactive oxygen species (ROS) and free radicals produced by cellular metabolism. Oxidative stress arises due to increased ROS production, instigated by an aberration in the equilibrium of adrenal hormones. Carotenoids possess the ability to scavenge ROS, thereby mitigating the deleterious effects of oxidative damage (122). Unbalanced levels of adrenal hormones can cause chronic inflammation, which is intimately related to oxidative stress. Anti-inflammatory characteristics found in carotenoids make them useful for controlling the inflammatory response. Carotenoids may indirectly lower oxidative stress levels linked to adrenal hormone imbalance by lowering inflammation (123). Unbalanced adrenal hormones can impact immunological performance and perhaps exacerbate oxidative damage. Through the stimulation of immune cell activity and the modulation of immunological responses, carotenoids have been demonstrated to support immune system function. An immune system that is in good health is better able to tolerate oxidative stress and minimize its effects (124). Although the direct effects of carotenoids on adrenal hormones have not been thoroughly investigated, they may indirectly influence the body’s hormonal balance. Certain hormones, particularly sex hormones, are partly produced and metabolized by carotenoids. Carotenoids may benefit adrenal hormone imbalance by promoting hormonal equilibrium, potentially lowering oxidative stress (125). When combined with other antioxidants like vitamins E and C, carotenoids can offer more antioxidant protection (126).

Depression is significantly influenced by carotenoid-cleaving enzymes, which are involved in the metabolism of carotenoids. It should be noted that the oxidative degradation of carotenoids, facilitated by carotenoid oxygenases, results in the formation of apocarotenoids. Retinal, retinol, retinoic acid, and abscisic acid are examples of apocarotenoids. By hyperactivating the hypothalamic-pituitary-adrenal (HPA) axis, retinoic acid, the active form of vitamin A, has been related to depressed behavior. Retinoic acid can cause suicide in sensitive people (127, 128). According to a study, eating foods high in carotene and vitamin C is linked to less severe depressive symptoms (129). Lower carotenoid levels may also be a result of bad eating habits linked to obesity and overweight, which have been linked to an enhanced risk of depression due to inflammation or HPA axis dysregulation (10).

### 6.4 Selenium against adrenal hormone imbalance-associated oxidative stress

Selenium is an indispensable trace mineral that serves as a potent antioxidant in the body. It exerts a protective effect on cellular structures by synergistic interactions with other antioxidants, such as vitamin E, thereby mitigating the deleterious impact of free radicals and oxidative stress-induced damage (130). Numerous antioxidant enzymes, such as glutathione peroxidase, which works to scavenge free radicals and lessen oxidative cell damage, require selenium as a cofactor. Free radicals can damage cells and cause chronic diseases like cancer, heart disease, and neurological disorders (131). By scavenging these free radicals, selenium reduces oxidative stress. In order to maintain a strong immune system, selenium is essential. It aids in controlling immunological responses, improves immune cell performance, and encourages the formation of antibodies (132). The synthesis and metabolism of thyroid hormones depend on selenium. It converts inactive thyroid hormone (T4) into active thyroid hormone (T3) to sustain proper thyroid function. Selenium has been investigated for its potential role in lowering the risk of specific cancers, including skin, lung, prostate, and colorectal cancers (133). As an antioxidant, it can aid in preventing DNA damage to cells and stop the development of cancer cells. By lowering oxidative stress, enhancing blood vessel function, and reducing inflammation, selenium may benefit heart health. These outcomes may assist in reducing the risk of cardiovascular conditions, such as heart disease and stroke. The health of the male reproductive system depends on selenium. It contributes to sperm production and aids in preserving the sperm cells’ structural integrity. It has been demonstrated that selenium supplementation enhances sperm motility and lessens sperm DNA damage (134). The recommended daily selenium intake varies depending on various factors, such as age, gender, and specific medical conditions. The recommended daily intake (RDA) for adults is about 55 micrograms per day. It is crucial to remember that consuming too much selenium can be hazardous, so stick to the recommended dosages. Brazil nuts, organ meats like liver and kidney, whole grains, eggs, and poultry are all excellent sources of selenium (135).

Selenium’s antioxidant capabilities and function in promoting the activity of antioxidant enzymes may have implications for lowering oxidative stress, even though there is little direct study on its impact on adrenal hormone imbalance-related oxidative stress (136).

Several antioxidant enzymes, such as thioredoxin reductases and glutathione peroxidases, require selenium in order to function. These enzymes are essential for scavenging ROS and guarding cells against oxidative damage. Increased ROS formation from adrenal hormone imbalance causes oxidative stress. To strengthen the body’s defence against oxidative stress, selenium aids in activating these antioxidant enzymes (137). The formation of glutathione, a potent antioxidant and detoxifying molecule, requires selenium. Glutathione is a key component of cellular antioxidant defence mechanisms and aids in the reduction of oxidative stress. Selenium shortage might hinder the production of glutathione, thereby aggravating the oxidative stress brought on by an imbalance in adrenal hormones. The generation and function of glutathione are supported by adequate selenium levels, promoting antioxidant defence (138). Unbalanced adrenal hormones can impact immunological performance and perhaps exacerbate oxidative damage. Immunomodulatory characteristics of selenium are well recognized, and it supports healthy immune system operation. A healthy immune system can better manage oxidative stress and lessen its harmful effects (139). Thyroid hormone metabolism, which is closely related to the control of adrenal hormones, involves selenium. The thyroid and hormonal equilibrium in the body may be supported by maintaining healthy selenium levels. Selenium may indirectly help to reduce the oxidative stress brought on by the imbalance of adrenal hormones by encouraging hormonal equilibrium (140).

Endocrine components of the “fight or flight” stress response include the hypothalamic-pituitary-adrenal (HPA) axis (**Figure 4**) (141). Corticotropin-releasing hormone (CRH) from the hypothalamus causes the anterior pituitary to release ACTH in response to stress. The production of corticosteroids, including the glucocorticoid class of stress hormones, is then triggered by ACTH acting on the adrenal gland. Almost every tissue in the body contains the GC receptor (GCR) (142). Because of their ability to reduce inflammation, GCs are frequently given for a wide range of ailments and diseases (143). Selenium appears to have a significant protective effect against the harm and dysfunction brought on by excessive activation of the HPA axis. Our research group has conducted a recent assessment of the progress made in investigating the relationship that has been extensively studied in the brain using rodent models in recent years (144).

A comprehensive investigation on porcine subjects has yielded noteworthy findings about the impact of selenium insufficiency on the antioxidant capacity and subsequent induction of oxidative stress within adrenal tissue. This phenomenon has been observed to occur through the mediation of the toll-like receptor 4 (TLR4)/NF-kB pathway. This observation contributes to the expanding body of evidence regarding the potential involvement of selenium in modulating the physiological functions of the adrenal gland (145). The observed correlation between selenium deficiency and reduced levels of miR-30d-R_1, a microRNA (miRNA) known for its inhibitory effect on TLR4 expression, implies a potential link between the dysregulation of the TLR4/NF-kB pathway and the onset of inflammatory processes (146). It is noteworthy to observe that the overexpression of TLR4 in human adrenocortical cells resulted in a reduction in the production of cortisol and aldosterone (147). Consequently, selenium has the potential to facilitate the functioning of the HPA axis through its ability to induce a mechanism of downregulation of TLR4 miRNA, thereby promoting the synthesis of adrenal steroids. The observed phenomenon of reduced corticosterone secretion due to selenium deficiency can be attributed to the blunting of the adrenal response to ACTH (139). The involvement of selenoproteins in the development of the HPA axis represents a captivating correlation between selenium and the physiological reaction to stress. During the developmental process of neuroendocrine cells, an intriguing observation was made about activating the Selenot gene in the adrenal medulla (148).

### 6.5 Zinc against adrenal hormone imbalance-associated oxidative stress

The immune system, cell division, and growth are just a few of the basic activities in the body that zinc is crucial for. Despite its relative lack of recognition as an antioxidant, zinc exhibits antioxidant properties and plays a crucial role in bolstering the body’s overarching antioxidant defense mechanisms (149). By scavenging damaging free radicals, zinc functions as an antioxidant to help protect cells from oxidative stress. Free radicals are unstable molecules that can harm cells and speed up the aging process. They also have a role in several disorders. Zinc is an antioxidant that helps to stabilize these free radicals and stop them from doing any harm. In addition to having antioxidant qualities, zinc also helps several enzymes involved in antioxidant defence systems function. It is an essential part of the enzyme SOD, which assists in converting superoxide radicals into less dangerous molecules (150). Additionally, zinc helps in the production of metallothionein, a protein that helps control metal levels and protects against oxidative damage. Enriching zinc consumption is crucial for the body to have a healthy antioxidant system. Oysters, red meat, chicken, beans, nuts, and whole grains are healthy food sources of zinc. Additional zinc supplements are available, which may be advantageous for people with specific health issues or zinc deficiency (151).

Zinc’s antioxidant capabilities and involvement in hormonal balance may have implications for lowering oxidative stress, even though there is little direct study on its effects on adrenal hormone imbalance-related oxidative damage (150).

Superoxide dismutase and catalase are two antioxidant enzymes that utilize zinc as a cofactor. These enzymes aid in reducing oxidative stress and neutralizing reactive oxygen species (ROS). Unbalanced levels of adrenal hormones can increase the generation of ROS, which increases the risk of oxidative damage. The body’s defence against oxidative stress is aided by zinc because of its role in the functioning of antioxidant enzymes (152). The synthesis, secretion, and metabolism of numerous hormones, particularly adrenal hormones, depend heavily on zinc. The proper production and control of these hormones can be hampered by an imbalance in the adrenal hormones. Zinc may indirectly lessen the oxidative stress brought on by an imbalance in adrenal hormones by promoting hormonal equilibrium (153). Unbalanced levels of adrenal hormones might affect how well the immune system works, thereby increasing oxidative stress. Zinc is important for immune system health and influences the growth and functioning of immune cells. A healthy immune system can better manage oxidative stress and lessen its harmful effects (154). Zinc aids in DNA repair processes and promotes healthy cellular operation. DNA can be harmed by oxidative stress, which can also harm other biological components. Zinc’s role in DNA repair promotes cellular health by preserving the integrity of genetic information and minimizing damage brought on by oxidative stress (155). Inflammation, which is strongly related to oxidative stress, can be brought on by a chronic adrenal hormone imbalance. The anti-inflammatory qualities of zinc make it possible to control the inflammatory response. Zinc may indirectly help lower oxidative stress levels linked to an imbalance in adrenal hormones by reducing inflammation (156).

Additionally, research has shown that cortisol impacts micronutrient metabolism, particularly magnesium, zinc, and selenium. Cortisol increases the expression of genes for metallothionein and ZIP-14, which accumulate zinc in the liver and adipose tissue, promoting hypozincemia in obese people (157).

Morais et al. (158) performed a correlation analysis to determine if cortisol affected zinc, magnesium, and selenium homeostasis in study participants. The plasma and erythrocyte zinc levels did not correlate with urine cortisol levels. Cortisol/cortisone ratio and erythrocyte zinc levels also correlated negatively. This study found hypozincemia in obese women due to elevated cortisol levels, which promote the production of metallothionein and ZIP-14 genes.

### 6.6 Polyphenols against adrenal hormone imbalance-associated oxidative stress

A class of naturally occurring substances called polyphenols can be found in a wide range of plant-based foods, such as fruits, vegetables, whole grains, herbs, and spices. Since they are recognized for having antioxidant capabilities, they can aid in preventing free radical damage to the body’s cells. Polyphenols are dietary antioxidants important for preserving general health and preventing chronic disorders (159).

By scavenging free radicals, polyphenols function as powerful antioxidants. Free radicals, characterized by their inherent instability, possess the capacity to inflict cellular damage and contribute significantly to the pathogenesis of various medical conditions, including but not limited to cancer, cardiovascular illnesses, and neurodegenerative disorders (160). By scavenging free radicals, polyphenols function as powerful antioxidants. Free radicals are unsteady molecules that can harm cells and play a role in the emergence of a number of illnesses, such as cancer, heart disease, and neurological disorders (161). Polyphenol-rich diets may have several positive health effects. Reducing inflammation, preventing cardiovascular disease, promoting brain health, boosting immunological function, and maybe lowering the risk of some cancers are a few of these (160). Inflammatory pathways in the body have been proven to be modulated by polyphenols, which help to lessen chronic inflammation, which is linked to a number of diseases like heart disease, obesity, and some types of cancer. Polyphenols may have biological effects besides their antioxidant action, such as encouraging good gut bacteria, enhancing blood sugar regulation, and promoting healthy aging processes (161).

Fruits, vegetables, tea, coffee, and cocoa are just a few examples of plant-based meals rich in polyphenols, a broad set of substances. Due to their anti-inflammatory and antioxidant capabilities, which may help lower oxidative stress and enhance general health, they have attracted much interest. While there is little direct evidence on how polyphenols affect the oxidative stress brought on by adrenal hormone imbalance, their antioxidant and anti-inflammatory properties may have implications for reducing oxidative stress (162).

Strong antioxidants, polyphenols can trap and deactivate free radicals and reactive oxygen species (ROS) produced by oxidative stress. Unbalanced levels of adrenal hormones can increase the generation of ROS, which increases the risk of oxidative damage. By quenching these detrimental chemicals and shielding cells from oxidative damage, polyphenols can help decrease oxidative stress (160). Inflammation, which is strongly related to oxidative stress, can be brought on by a chronic adrenal hormone imbalance. The inflammatory response can be modulated by polyphenols, which have anti-inflammatory effects. Polyphenols may indirectly help lower oxidative stress levels linked to an imbalance in adrenal hormones by reducing inflammation (163). Polyphenols have been demonstrated to alter the body’s signalling systems and hormone levels. Polyphenols may have indirect impacts on hormone control even though their direct effects on adrenal hormones are not fully understood. Polyphenols may potentially assist in lowering oxidative stress linked to adrenal hormone imbalance by supporting hormonal equilibrium (164). Unbalanced levels of the adrenal hormones can cause mitochondria to malfunction and produce more ROS. It has been demonstrated that polyphenols enhance mitochondrial function and defend them against oxidative damage. Polyphenols support cellular health by protecting mitochondrial health and lowering oxidative stress (163). Superoxide dismutase (SOD) and catalase are two examples of endogenous antioxidant enzymes that can be stimulated by polyphenols. These enzymes are essential for reducing ROS and preserving redox equilibrium. Polyphenols may offer extra defence against oxidative stress related to an imbalance in adrenal hormones by increasing the activity of these antioxidant enzymes (165).

Polyphenols activate the redox-sensitive transcription factor nuclear factor erythroid 2-related factor-2 (Nrf2) (166). Contrarily, research suggests that polyphenols can modify the glucocorticoid receptor’s (GR) activity. In fact, GR and FK506 binding protein 5 (FKBP5) expression can be changed by the polyphenolic flavonoid icariin, which enhances GR stability and lessens GR sensitivity to GC *in vivo* (167). More research is required in this area since manipulation of the GR regulatory system is currently an intriguing target for the treatment of stress-related illnesses (168).

### 6.7 Coenzyme Q10 against adrenal hormone imbalance-associated oxidative stress

A naturally occurring substance in the body is coenzyme Q10 (CoQ10). CoQ10 plays a pivotal role in generating adenosine triphosphate (ATP), which serves as the primary energy source for cells. Additionally, CoQ10 performs the role of an antioxidant, assisting in preventing cell deterioration brought on by harmful molecules known as free radicals. CoQ10 acts as an antioxidant to combat free radicals and stop oxidative stress, which can cause cellular damage and be a factor in several health issues (169). In order to stabilize free radicals and lessen their potential for harm, it donates electrons. Small levels of coenzyme Q10 are included in some meals, including meat, fish, and whole grains. However, CoQ10 production by the body tends to decrease with aging, and some diseases or drugs might further lower its levels. CoQ10 supplements are therefore offered to support optimal levels of this substance. CoQ10 has been researched for its possible health advantages in several illnesses, even though it is primarily known for its role in energy production and as an antioxidant (170).

Despite the limited body of research investigating the specific impact of CoQ10 on oxidative stress resulting from imbalances in adrenal hormones, it’s inherent antioxidant properties and involvement in cellular energy metabolism suggest potential efficacy in mitigating oxidative stress (171).

CoQ10 is a powerful antioxidant that protects cells from the damage that free radicals and reactive oxygen species (ROS) cause when they combine with oxygen. Dysregulation of adrenal hormones can lead to increased production of ROS, hence contributing to oxidative stress. CoQ10 helps eliminate these harmful molecules, which lowers oxidative stress and keeps the health of cells (172). CoQ10 is an important part of the electron transport chain, which is a process that helps cells make energy (ATP). A lack of adrenal hormones can change how cells use energy, which can increase oxidative stress. CoQ10 may help restore cellular balance and lower oxidative stress by helping cells make energy more efficiently (173). CoQ10 can make antioxidants like vitamin E, vitamin C, and glutathione, which are important parts of the body’s antioxidant defence system, from scratch. By regenerating and reusing these antioxidants, CoQ10 makes them more effective at fighting oxidative stress caused by an imbalance in adrenal hormones (174). A lack of adrenal hormones that lasts for a long time can cause inflammation, which is linked to oxidative stress. CoQ10 has anti-inflammatory qualities and can help control the way the body reacts to inflammation. CoQ10 may indirectly help reduce oxidative stress by reducing inflammation (51). CoQ10 keeps cell parts, like cell walls and mitochondria, from getting damaged by oxidation. Unbalanced adrenal hormones can cause oxidative stress in cells, which can damage the structures of cells. CoQ10 helps keep cell walls and mitochondria working well, which reduces damage caused by oxidative stress (175).

When it comes to disorders with the pituitary and adrenal glands, there is proof of mitochondrial dysfunction in people with Cushing’s syndrome. This is shown in respiratory chain complex enzyme activity (176) and oxidative stress (measured by total antioxidant capacity and plasma 15-F2t-isoprostane) (177). However, there haven’t been many studies on how CoQ10 affects pituitary and adrenal function in endocrine therapy. Some pituitary/adrenal problems may be associated with low levels of CoQ10 in the blood, according to preliminary investigations (178). Plasma CoQ10 levels were evaluated in six patients with ACTH-dependent adrenal hyperplasia, 19 with secondary solitary hypoadrenalism, and 19 with concurrent hypothyroidism (multiple pituitary deficits). Compared to numerous pituitary deficits and ACTH-dependent adrenal hyperplasia, CoQ10 levels were considerably lower in secondary isolated hypoadrenalism. Patients with acromegaly apparently have lower plasma CoQ10 levels (179).

### 6.8 Probiotics against adrenal hormone imbalance-associated oxidative stress

Typically, probiotics are not regarded as a direct source of antioxidants. However, some probiotic strains have been demonstrated to have indirect antioxidant benefits or to be able to improve the body’s antioxidant state via a variety of mechanisms (180).

A healthy, diversified gut flora, which is important for the digestion of food components, can be maintained with the use of probiotics. Some helpful bacteria in the stomach can accelerate the breakdown and creation of bioactive molecules with antioxidant capabilities from dietary antioxidants, such as polyphenols found in fruits and vegetables. The availability and potency of antioxidants in the body may increase as a result of this metabolic activity (181). It has been demonstrated that probiotics can indirectly lower oxidative stress by enhancing gut health and lowering inflammation. Probiotics can assist in maintaining gut barrier function, preventing the transfer of risky bacteria and endotoxins into the bloodstream, and reducing systemic inflammation by supporting a healthy gut microbiota. This can then support general antioxidant defence mechanisms and minimize oxidative stress (182). Probiotics can increase the body’s natural synthesis and activity of endogenous antioxidants. For instance, it has been discovered that specific probiotic strains enhance the production of glutathione, a potent antioxidant that aids in neutralizing free radicals and the defence of cells against oxidative damage (180). Probiotics can interact with intestinal mucosal cells and other tissues to enhance their antioxidant defences. They can affect cellular signaling pathways that produce antioxidant enzymes like SOD and catalase, which remove free radicals and minimize oxidative stress (183).

Probiotics, known as beneficial microorganisms that promote a healthy gut microbiota, have exhibited promising potential in modulating the equilibrium of adrenal hormones and mitigating challenges associated with oxidative stress. The interaction between the gut microbiota and the adrenal glands is reciprocal, which means that each can have an impact on the other’s operation. Dysbiosis, a condition characterized by perturbations in the composition and function of the gastrointestinal microbiota, has been implicated in various pathological states, encompassing abnormalities of adrenal hormone levels and elevated oxidative stress (184).

**Figure 5:**
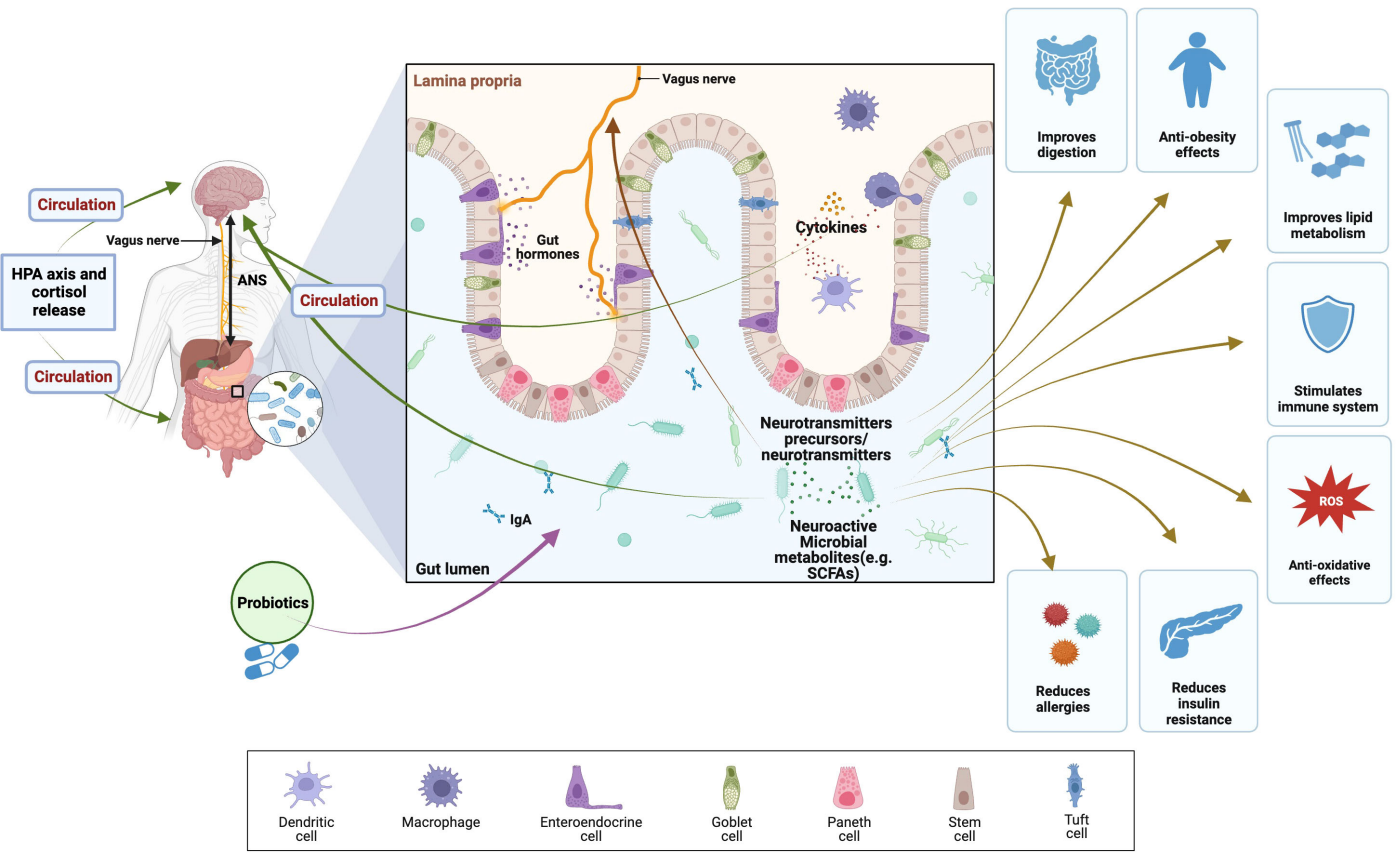
Health benefits of probiotics and their effects on brain, gut, and microbiome (BGM) axis modulating of HPA axis and cortisol release. The BGM axis network of routes that facilitate the exchange of information and signals encompasses neuronal elements (vagus nerve, neurotransmitters, and enteric nervous system), the HPA axis, and stress hormones like cortisol. Furthermore, immune mechanisms, specifically cytokines, contribute to this complex interplay. (SCFAs), Short-chain fatty acids; (ANS), autonomic nervous system; (ROS), reactive oxygen species; (HPA axis), Hypothalamic–pituitary–adrenal axis. The figure was produced with BioRender (Biorender.com; accessed on 30^th^ Oct 2023).

Probiotics support a balanced population of good bacteria in the gut, enhancing overall gut health (**Figure 5**). Better nutrient absorption, especially of antioxidants and other vital micronutrients necessary for preventing oxidative stress, is made possible by a healthy gut lining (185). Probiotic supplementation has been shown to exert a modulatory effect on the body’s inflammatory and immunological responses. Chronic inflammation can disturb the equilibrium of the adrenal hormones and is linked to increased oxidative stress. Probiotics may indirectly aid adrenal gland function and lessen oxidative stress by lowering inflammation (186). In the gut, some probiotic strains can create antioxidants. For instance, it has been demonstrated that certain *Lactobacillus* and *Bifidobacterium* species produce antioxidants like glutathione, which can help mitigate oxidative stress and protect against cell damage (187). Probiotics can convert polyphenols present in fruits and vegetables, for example, into bioactive compounds with antioxidant properties. This transformation engenders heightened accessibility and potency of antioxidants, thereby fostering equilibrium in adrenal hormone levels and mitigating the impact of oxidative stress (180). Probiotics can influence the body’s response to stress and help regulate cortisol levels. By promoting a balanced stress response, probiotics may assist in regulating adrenal hormone production and mitigating the harmful effects of stress-induced oxidative stress (188).

Bidirectional connections within the brain-gut-microbiome (BGM) axis have been demonstrated in several preclinical and clinical research studies (189, 190). The communication between gut microbes and the central nervous system is facilitated through a complex network of interconnected channels, which encompass the nervous, endocrine, and immune signaling mechanisms. The brain possesses the capability to exert influence on the structural and functional organization of the gut microbiota. This influence is primarily mediated by the autonomic nervous system (ANS), which regulates various aspects, including gut permeability, regional gut motility, intestinal transit and secretion. The HPA axis operates under the fundamental mechanism of negative feedback and assumes a pivotal function in eliciting the body’s stress response and governing various physiological processes, encompassing digestion, immune system functionality, and energy equilibrium (191). Stress hormones induce the disruption of tight junctions, consequently leading to increased permeability of the intestinal barrier (192). Dietary probiotic supplementation has been shown to offer potential alleviation of the HPA axis response to acute stress (193). For example, the administration of a probiotic treatment containing *L. farciminis* in a murine model has been reported to effectively mitigate the stress-induced hyperpermeability, endotoxemia, and, thus, ameliorating the stress response of the HPA axis.

Moreover, recent research findings have indicated that using probiotics and prebiotics, which serve as agents that regulate the composition of the gastrointestinal microbiota, may offer potential advantages in mitigating the manifestations of stress-related infertility (194). The beneficial effects of probiotics on infertility associated with stress have been attributed to various mechanisms, such as regulating the HPA axis, modulation of the immune response, and restoring microbial homeostasis (184). The HPA axis plays a pivotal role in maintaining reproductive health by governing the intricate regulation of the stress response. The HPA axis can be disrupted by stress, resulting in variations in cortisol levels, the primary stress hormone (49). Multiple studies have provided evidence indicating that probiotics possess the ability to modulate the HPA axis and reduce cortisol levels, thereby alleviating the adverse impact of stress on reproductive health (194). Under stressful circumstances, *Lactobacillus casei* strain Shirota (LcS) may prevent cortisol hypersecretion and physical symptoms, possibly through reducing stress reactivity in the paraventricular nucleus (PVN) and vagal afferent signaling to the brain (195). According to Nasri et al. (196), selenium and probiotic co-administration to women with polycystic ovary syndrome (PCOS) reduced modified Ferriman Gallwey (mF-G) scores and total testosterone.

**Figure 6:**
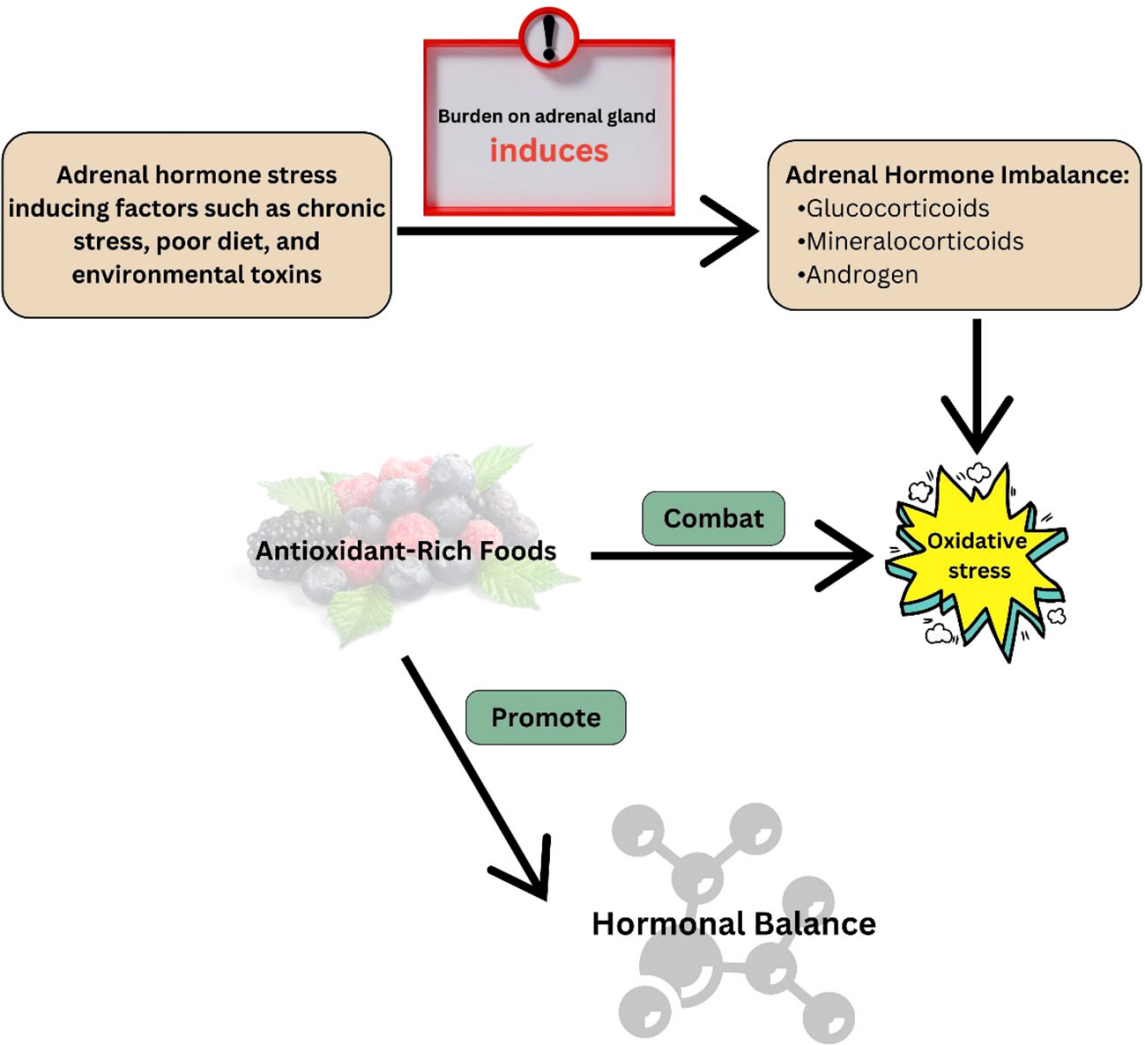
Role of nutritional antioxidants in alleviating adrenal hormone imbalance.

The role of nutritional antioxidants in alleviating adrenal hormone imbalance is depicted in (**Figure 6**) (**Table 2**).

**Table 2:** The role of nutritional antioxidants in Adrenal hormone function.

Nutritional Antioxidant	Role in Adrenal Hormone Function	Reference
Vitamin C	- Supports the production of androgens, glucocorticoids, and mineralocorticoids - Serves as a cofactor in the process by which cholesterol is transformed into pregnenolone	(103)
Vitamin E	- Protects adrenal cells from oxidative stress - Possibly plays a function in regulating cortisol levels	(197)
Carotenoids	- Carotenoids contained in several foods, beta-carotene and lycopene, act as antioxidants - Aiding in the reduction of oxidative stress in the adrenal glands	(10)
Selenium	Important in the synthesis of selenoproteins such as glutathione peroxidase, which protects adrenal cells from oxidative damage.	(144)
Zinc	- Zinc is required for the synthesis, release, and general function of adrenal hormones - As an antioxidant, it protects cells from oxidative stress.	(198)
Polyphenols	Reduce oxidative damage and inflammation in the adrenal glands to help with adrenal hormone balance.	(161)
Coenzyme Q10	- Plays a critical function in the cellular energy production process - Supports the overall function of the adrenal glands and may lessen oxidative stress.	(179)
Probiotics	- Indirectly altering adrenal hormone balance and encouraging optimal function by mitigating oxidative stress and inflammation.	(199)

## 7 Search strategy

This review paper’s search strategy included a thorough study of scientific databases such as PubMed, Scopus, Google Scholar, and Web of Science, utilizing a combination of keywords and controlled vocabulary terms. The most commonly used terms were “oxidative stress,” “oxidative stress,” “nutritional antioxidants,” “reactive oxygen species,” and “adrenal hormone imbalance.” The search was refined using Boolean operators (AND, OR) to ensure relevance to the review’s focus on the potential of nutritional antioxidants against oxidative stress linked with adrenal hormone imbalance. Furthermore, specific terms relating to adrenal gland function, such as “glucocorticoids,” “mineralocorticoids,” “androgens,” and “adrenal hormone disorders” such as “adrenal insufficiency,” “Cushing’s syndrome,” and “adrenal tumors,” were added to collect relevant literature. The search included experimental and clinical investigations, as well as review papers, to provide a full picture of the current level of knowledge on the topic. The inclusion criteria included publications published in the previous decade, and the search approach was iterative, with continual refinement based on the identified literature until a thorough selection of relevant studies was obtained.

## 8 Conclusions

In conclusion, the effectiveness of nutritional antioxidants in combating oxidative stress caused by adrenal hormone imbalance is undeniable. Antioxidants, such as vitamin C, vitamin E, carotenoids, selenium, zinc, polyphenols, coenzyme Q10, and probiotics, play vital roles in mitigating the negative effects of oxidative stress on adrenal hormone balance. By mitigating dangerous free radicals and reducing oxidative damage, these antioxidants can aid in the restoration and maintenance of adrenal hormone function. In addition, the article discusses adrenal hormone abnormalities such as adrenal insufficiency, Cushing’s syndrome, and adrenal tumors. The findings imply that utilizing the efficacy of dietary antioxidants may offer therapeutic approaches for alleviating oxidative stress associated with adrenal hormone abnormalities, opening up new areas for investigation.

## Author contributions

AnP: Data curation, Investigation, Methodology, Writing – original draft, Writing – review & editing. DB: Data curation, Formal Analysis, Resources, Software, Writing – review & editing. VY: Conceptualization, Methodology, Supervision, Validation, Writing – review & editing. K-YL: Resources, Software, Supervision, Validation, Writing – review & editing. AsP: Formal Analysis, Investigation, Validation, Visualization, Writing – original draft, Writing – review & editing. DS: Conceptualization, Formal Analysis, Funding acquisition, Visualization, Writing – original draft, Writing – review & editing.
